# A Quality, Size and Time Assessment of the Binarization of Documents Photographed by Smartphones

**DOI:** 10.3390/jimaging9020041

**Published:** 2023-02-13

**Authors:** Rodrigo Bernardino, Rafael Dueire Lins, Ricardo da Silva Barboza

**Affiliations:** 1 Centro de Informática, Universidade Federal de Pernambuco, Recife 50.670-901, PE, Brazil; 2 Departamento de Computação, Universidade Federal Rural de Pernambuco, Recife 55.815-060, PE, Brazil; 3 Coordenação de Engenharia da Computação, Escola Superior de Tecnologia, Universidade do Estado do Amazonas, Manaus 69.410-000, AM, Brazil

**Keywords:** document binarization, photographed documents, DIB-dataset, smartphone, binarization algorithms

## Abstract

Smartphones with an in-built camera are omnipresent today in the life of over eighty percent of the world’s population. They are very often used to photograph documents. Document binarization is a key process in many document processing platforms. This paper assesses the quality, file size and time performance of sixty-eight binarization algorithms using five different versions of the input images. The evaluation dataset is composed of deskjet, laser and offset printed documents, photographed using six widely-used mobile devices with the strobe flash off and on, under two different angles and four shots with small variations in the position. Besides that, this paper also pinpoints the algorithms per device that may provide the best visual quality-time, document transcription accuracy-time, and size-time trade-offs. Furthermore, an indication is also given on the “overall winner” that would be the algorithm of choice if one has to use one algorithm for a smartphone-embedded application.

## 1. Introduction

The current number of smartphone users in the world today is over 6.6 billion (Source: https://www.bankmycell.com/blog/how-many-phones-are-in-the-world, last visited on 29 December 2022), which means that over 83% of the world’s population owns a smartphone. The omnipresence of smartphones with in-built cameras made most people (91%) take photos with smartphones, while only 7% use digital cameras or tablets (2%). According to that same website, the forecast figures by Ericsson and the Radicati Group, that percentage is expected to grow from 91% in 2022 to 94% in 2026. Consumers see the quality of the camera as a key factor in choosing a smartphone model. Thus, since cameras became the most significant selling point on smartphones, manufacturers have been putting much effort into improving their quality. At first, they paid more attention to the amount of megapixels a smartphone camera could pack. In the last few years, smartphone manufacturers have opted to add more cameras to their phones to improve photo quality and optical zoom functionality while keeping the device thin. Each camera has a lens that can yield either a wide shot or a zoomed-in shot. Some phones have additional black and white cameras for increased light sensitivity, while others offer depth information. Data from the different cameras can be combined into a clear photo with seemingly shallow depth-of-field and good low-light capability.

Taking photos of documents with smartphone cameras, an attitude that started almost two decades ago [1,2,3,4], became of widespread use today. It is extremely simple and saves photocopying costs, allowing the document image to be easily stored and shared using computer networks. However, smartphone cameras were made to take family and landscape photos or make videos of such subjects and were not targeted at document image acquisition. Smartphone document images have several problems that bring challenges to processing them. The resolution and illumination are uneven, there are perspective distortions, and often the interference of external light sources [4]. Even the in-built strobe flash may add further difficulties if activated by the user or automatically. Besides all that, the standard file format used by smartphone cameras to save the images is jpeg, which inserts the jpeg noise [5], a light white noise added to prevent two pixels of the same color from appearing next to each other. This noise makes the final image more pleasant to the human eye glancing at a landscape or family photo, but it also means a loss in sharpness in a document image, bringing difficulties to any further processing.

The conversion of a color image into its black-and-white version is called thresholding or binarization. It is a key step in the pipeline of many document processing systems, including document content recovery [6]. The binarization of scanned document images is far from being a simple task as the physical noises [7], such as paper aging, stains, fungi, folding marks, etc., and back-to-front interference [8] increase the complexity of the task. In the case of scanned documents, some recent document binarization competitions [9,10] show that no single binarization algorithm is efficient for all types of text document images. Their performance depends on a wide number of factors, from the digitalization device, image resolution, the kind of physical noises in the document [7], the way the document was printed, typed or handwritten, the age of the document, etc. Besides that, those competitions showed that the time complexity of the algorithms also varies widely, making some of them impossible to be used in any document processing pipeline. Thus, instead of having an overall best, those competitions pointed out the top quality-time algorithms in several categories of documents.

The binarization of photographed documents is far more complex than scanned ones and, as already mentioned above, the resolution and illumination are uneven, among several other problems. Besides that, each smartphone model has different camera features. The first competition to assess the quality and time of the binarization of smartphone camera-acquired text documents, the type of document that is most often photographed, comparing new algorithms with previously published and more classical ones was [11]. In 2021, that same competition occurred with several new competitors and devices [12].

Binary images also are much smaller than their color counterparts, thus their use may save storage space and computer bandwidth [13]. This means that assessing the resulting image file size using a lossless compression scheme is also relevant for comparison among binarization algorithms. Besides that, the binary image may be the key for generating colored synthetic images, which are visually indistinguishable from the original document whenever printed or visualized on a screen [14]. Run-length encoding [15] the sequences of black and white pixels is the key to several schemes for compressing monochromatic images. Suppose the binarization process leaves salt-end-pepper noise in the final image, sometimes imperceptible to the human eye. In that case, that noise will break the sequence of similar pixels, degrading the performance of the image compression scheme. Indirectly, that can also be observed as a measure of the quality of the monochromatic image. The third venue [16] of the ACM DocEng Competition on the binarization of photographed documents assessed five new and sixty-four algorithms, and it was possibly the first time the size of the monochromatic image was considered in the assessment of the binarization algorithms, ever.

Reference [17] shows that feeding the binarization algorithms with the different red, green and blue (RGB) channels, instead of the whole image, may yield a better quality two-tone image, besides saving processing time. This paper largely widens the scope of [16] as, due to the restricted time to produce the final report, it was impossible to process and assess the quality, time, and file size of the almost 350 binarization schemes. Besides that, also due to processing time limitations, the file-size assessment was ranked based on the quality of the optical character recognition (OCR) transcription based on the Levenshtein distance to the ground-truth text. In contrast, here, one ranks the algorithms based on a new image quality measure introduced here, possibly a more adequate measure.

The recent paper [18] presents a methodology to pinpoint which binarization algorithm would provide the best quality-time trade-off either for printing or for OCR-transcription. It also proposes an overall winner if one would choose one single algorithm capable of being embedded in applications in a smartphone model. The present paper also makes such choices for each of the smartphones assessed.

## 2. Materials and Methods

Six different models of smartphones from three different manufacturers, widely used today, were used in this assessment. Their camera specification is described on Table 1. Their in-built strobe flash was set on and off to acquire images of offset, laser, and deskjet printed text documents photographed at four shots with small variations in the position and moments, to allow for different interfering light sources. The document images captured with the six devices were grouped into two separate datasets:

- **Dataset 1:** created for 2022 DocEng contest [16], the photos were taken with devices Samsung N10+ (Note 10+) (Samsung Electronics, Suwon-si, South Korea) and Samsung S21U (Ultra 5G) (Samsung Electronics, Suwon-si, South Korea). It has challenging images with natural and artificial light sources and with strong shadows;
- **Dataset 2:** created for 2021 DocEng contest [19], the photos were taken with devices Motorola G9 (Motorola Mobility, Chicago, IL, USA), Samsung A10S (Samsung Electronics, Suwon-si, South Korea), Samsung S20 (Samsung Electronics, Suwon-si, South Korea) and Apple iPhone SE 2 (Apple Inc., Cupertino, CA, USA). It also has challenging images, but they are less complex than Dataset 1.

The test images were incorporated to the IAPR (International Association for Pattern Recognition) DIB - Document image binarization platform (https://dib.cin.ufpe.br, accessed on 17 January 2023)), which focuses on document binarization. It encompasses several datasets of document images of historical, bureaucratic, and ordinary documents, Which were handwritten, machine-typed, offset, laser, and ink-jet printed, both scanned and photographed, several of them with their corresponding ground-truth images. Besides being a document repository, the DIB-platform encompasses a synthetic document image generator, which allows the user to create over 5.5 million documents with different features. As already mentioned, reference [17] shows that binarization algorithms, in general, yield different quality images whenever fed with the color, gray-scale-converted, and R, G, and B-channels. Here, 68 classical and recently published binarization algorithms are fed with the five versions of the input image, totaling 340 different binarization schemes. The complete list of the algorithms used is presented in Table 2, along with a short description and the approach followed in each of them.

The quality of the final monochromatic image is the most important assessment criterion. Once one has the top-quality images, one may consider the mean size of the monochromatic files and the mean time elapsed by each of the assessed algorithms through the dataset. This paper proposes a novel quality measure for photographed document images called PL, it is a combination of the previously proposed $P_{err}$ [70] and $\left[L_{dist}\right]$ [11] measures. Two quality measures were used to evaluate the quality of the binarization algorithms: the $\left[L_{dist}\right]$ and the PL.

### 2.1. The Quality Measure of the Proportion of Pixels ($P_{err}$)

Assessing image quality of any kind is a challenging task. The quality of photographed documents is particularly hard to evaluate as the image resolution is uneven, it strongly depends on the features of the device, the distance between the document and the camera and it even suffers from perspective distortion. Creating a ground-truth (GT) binary image for each photographed document would require a non-viable paramount effort. An alternative method [70] was used: the paper sheet or book page is scanned at 300 dpi, binarized with several algorithms, visually inspected, and manually selected and retouched to provide the best possible binary image of that scanned document, which will generate the reference proportion of black pixels for that document image. The $P_{err}$ measure compares the proportion between the black-to-white pixels in the scanned and photographed binary documents, as described in Equation (1):

(1) $$P_{err}=abs\left(PB_{bin}-PB_{GT}\right),$$

where PB=100×(B/N) is the proportion of black pixels in the image, *B* is the total number of black pixels and *N* is the total number of pixels in the image. Thus, $PB_{bin}$ is the proportion of black pixels in the binary image and $PB_{GT}$ is the proportion of black pixels in the scanned ground-truth image.

**Figure 1:**
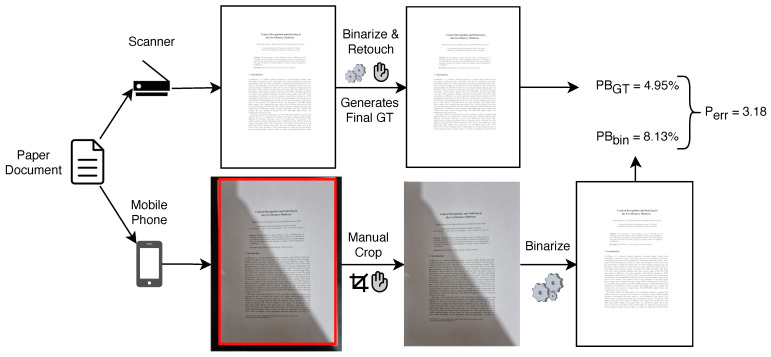
$P_{err}$ measure example (GT: ground-truth, bin: binary).

In order to provide a fair assessment, the photographed image must meet several requirements. The resolution of the output document photo must be close to 300 dpi (which correspond to the scanned one). To meet such a requirement, the camera should have around 12 Mpixel resolution and the document should fill nearly all the photographed image; the photo must be cropped to remove any reminding border. Here, the cropping is manually done, as the focus is to assess specifically the binarization algorithms. Figure 1 describes the preparation of the images and an example of $P_{err}$ calculation. The $P_{err}$ was used by the last DocEng contests [11,12,16] to evaluate the quality of the binary images for printing and human reading.

### 2.2. Normalized Levenshtein Distance ($\left[L_{dist}\right]$)

The second quality measure is the Optical Character Recognition (OCR) correctness rate measured by $\left[L_{dist}\right]$ [11], which is the Levenshtein [71] distance normalized by the number of characters in the text. Google Vision OCR was used to obtain the machine-transcribed text. It is important to note that Google Vision automatically detects the input language and applies post-processing based on dictionary, which cannot be deactivated. The Levenshtein distance, here denoted by $L_{dist}$, expresses the number of character insertion, deletion and replacements that would be necessary to convert the recognized text into the manually transcribed reference text for each image. Thus, the $L_{dist}$ depends on the length of the text and cannot be used to measure the performance across different documents as an absolute value. In [11], a normalized version of the $L_{dist}$ was proposed, calculated as:

(2) $$\left[L_{dist}\right]=\frac{\#char-L_{dist}}{\#char},$$

where #char is the number of characters in the reference text.

**Figure 2:**
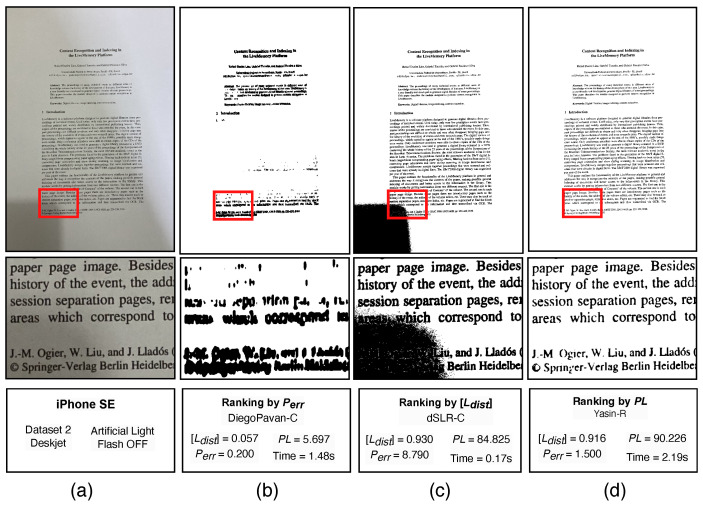
Comparison between different measures: PL, $\left[L_{dist}\right]$, $P_{err}$. For each case, the full image is shown on the top and an example region bellow, where the red boxes indicates the crop position for the example region. (**a**) Original image; (**b**) Ranking by $P_{err}$ only, DiegoPavan-C binarized image; (**c**) Ranking by $\left[L_{dist}\right]$ only, dSLR-C binarized image; (**d**) Ranking by PL measure, Yasin-R binarized image.

The DocEng 2022 binarization competition for photographed documents presented a new challenging dataset in which complex shaded areas were introduced. Although the $P_{err}$ quality measure worked well whenever the shaded area was more uniformly distributed, in those more complex multi-shaded documents, some algorithms may concentrate the pixels around some characters (e.g. by dilatation) while completely removing other parts of the document. This could generate an image that has the same proportion of black pixels as the ground-truth, a clear background with no evident noise, but its text is unreadable. Taking, for instance, an example image taken with Apple iPhone SE2 of a deskjet printed document with the strobe flash off (Figure 2a), the algorithm with the closest black pixel proportion would be DiegoPavan provided the original color image. The result is presented in Figure 2b. Note that even the remaining dilated letters are nearly unreadable, giving a $\left[L_{dist}\right]$ of nearly zero, meaning almost no text was transcribed. The $P_{err}$ close to zero means the proportion of black pixels is very close to the ground-truth.

If one ignores the $P_{err}$ and only sorts the results by $\left[L_{dist}\right]$, the most recommended algorithm would be dSLR, having the original color image as input. The result of such binarization is presented in Figure 2c for the same image. Nearly all the text was successfully transcribed ($\left[L_{dist}\right]$ close to 1.0), however, there is a large noisy area in the bottom-left corner, which only did not significantly affected the transcription due to the large margins of the document. Such a noise was generated by a shadow of the mobile phone and could not be detected by $\left[L_{dist}\right]$ measure, but checking $P_{err}$ it is clear that a large amount of noise is present. A printed document usually has nearly 5% of text pixels (in this image, it was 3.77%), thus a difference of 8.79 from the ground-truth is a large one. If one would want just to transcribe the text, it could be enough to use such an algorithm for that image; however, if the margins were smaller or the binarized document would be printed, such a large noise blurb would be unacceptable.

### 2.3. Pixel Proportion and Levenshtein Measure (PL)

In order to obtain the best OCR quality while providing visually pleasant human-readable binary document images, a new quality measure is proposed here:

(3) $$PL=\left[L_{dist}\right]\times \left(100-P_{err}\right).$$

Applying such a new measure to the already presented examples of document images would yield PL=5.69 for DiegoPavan-C and PL=84.82 for dSLR-C, while the best algorithm, according to the proposed quality measure, Yasin-R, yields PL=90.22. The corresponding image is presented in Figure 2d, and it has a better overall visual quality and OCR transcription rate, although the dSLR algorithm is an order of magnitude faster than the other two algorithms.

### 2.4. TIFF Group 4 Compression Rate ($CR_{G4}$)

This work also assessed the size of the monochromatic image files compressed using the Tag Image File Format Group 4 (TIFF_G4) with Run-length encoding (RLE), a new quality measure for monochromatic images recently introduced in [16]. Such a compression scheme is part of the Facsimile (FAX) recommendation and was implemented in most FAX systems at a time when transmitting resources were scarce. The TIFF_G4 file format is possibly the most efficient lossless compression scheme for binary images [5]. One central part of such an algorithm is to apply run-length encoding [15]. Thus, the less salt-and-pepper noise present in the binary image, the longer the sequences of the same color bits, yielding a smaller TIFF_G4 file, which claims for less bandwidth for network transmission and less storage space for archiving. The compression rate is denoted by $CR_{G4}$ and is calculated by:

(4) $$CR_{G4}=100\times \frac{S_{G4}}{S_{PNG}},$$

where $S_{G4}$ denotes the size of the compressed TIFF G4 file and $S_{PNG}$ is the size of the Portable Network Graphics (PNG) compressed file with compression level 4. It is important to remark that such a measure should be used not as an isolated quality measure, but only to re-rank the algorithms with the best PL, as it provides a secondary fine-grained quality measure.

### 2.5. Processing Time Evaluation

The viability of using a binarization algorithm in a document processing pipeline depends not only on the quality of the final image, but also on the processing time elapsed by the algorithm and the maximum amount of memory claimed during the process. To the best knowledge of the authors, the first assessment of binarization algorithms to take the average processing time into account was [9]. The assessed algorithms were implemented by their authors using several programming languages and operating systems, running in different platforms, thus the processing time figures presented here provide the order of magnitude of the time elapsed for binarizing the whole dataset. The training times for the AI-based algorithms were not computed. Two processing devices were used:

- **Device 1 (CPU algorithms):** Intel(R) Core(TM) i7-10750H CPU @ 2.60 GHz, with 32 GB RAM and a GPU GeForce GTX 1650 4 GB.
- **Device 2 (GPU algorithms):** Intel(R) Core(TM) i9-9900K CPU @ 3.60 GHz, with 64 GB RAM and a GPU NVIDIA GeForce RTX 2080 Ti 12 GB.

The algorithms were implemented using two operating systems and different programming languages for specific hardware platforms such as GPUs:

- **Device 1, Windows 10 (version 1909), Matlab (version 9.4):** Akbari_1, Akbari_2, Akbari_3, CLD, CNW, ElisaTV, Ergina-Global, Ergina-Local, Gattal, Ghosh, HBUT, Howe, iNICK, Jia-Shi, Lu-Su, Michalak, MO_1_, MO_2_, MO_3_, Yasin;
- **Device 1, Linux Pop!_OS (version 20.10):** Bataineh, Bernsen, Bradley, Calvo-Zaragoza, daSilva-Lins-Rocha, DiegoPavan, Huang, Intermodes, ISauvola, IsoData, Johannsen-Bille, Kapur-SW, Li-Tam, Mean, Mello-Lins, MinError, Minimum, Moments, Niblack, Nick, Otsu, Percentile, Pun, RenyEntropy, Sauvola, Shanbhag, Singh, Su-Lu, Triangle, Vahid22, WAN, Wolf, Wu-Lu, Yen, YinYang, YinYang21, YinYang22;
- **Device 2, Linux Pop!_OS (version 22.04):** DE-GAN, DeepOtsu, DilatedUNet, Doc-DLinkNet, Doc-UNet, DPLinkNet, HuangBCD, HuangUnet, Robin, Vahid, Yuleny.

The algorithms were executed on different operating systems (OS), but on the same hardware. For those that could be executed on both OS types, the processing times for each OS was measured and no significant difference was noticed. This is expected based on previous experimentation [11]. The mean processing time was used in the analysis. As already mentioned, the primary purpose is to provide the order of magnitude time of the processing time elapsed.

### 2.6. Quality, Space and Time Evaluation

**Figure 3:**
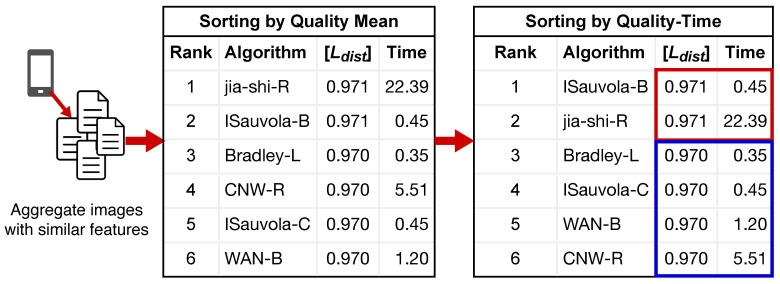
Example of ranking by the quality-time criteria. The algorithms are first sorted by quality ($\left[L_{dist}\right]$) and then by time. The red and blue boxes highlight that the first two algorithms have the same quality results and thus are sorted separately from the other four.

For each of the six devices studied, this paper assesses the performance of the 340 binarization schemes listed applied to photographed documents, with the strobe flash on and off, in two different ways:

1. **Best quality-time and compression:** applies the ranking by summation, followed by sorting by processing time, but clustering by device and observing the compression rate for the top-rated algorithms.
2. **Image-specific best quality-time:** makes use of PL and $\left[L_{dist}\right]$. The ranking is performed by first sorting according to the quality measure and when the quality results are the same, sorted by processing time. This is illustrated in Figure 3.

**Figure 4:**
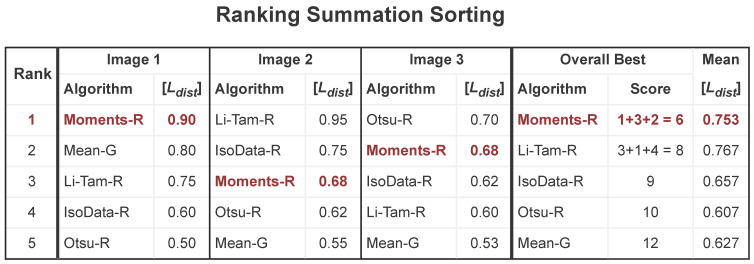
Example of sorting by the ranking summation criterion. The algorithm marked in red (Moments-R) is the overall best according to this criterion.

The ranking summation applied to binarization was first applied on the series of competitions Document Image Binarization Competition (DIBCO) [72] and has been then used in many subsequent competitions and assessments [9]. In Figure 4 a visual description of this criterion is presented. First, the algorithms are ranked in the context of each image individually, then the ranking position is summed up across the images, composing the score for each algorithm. The final ranking is determined by sorting the algorithms by the score, and the global mean of all images is presented to provide a quantitative overall ordering.

Sorting directly by the mean of the quality measure gives less precise results, as one seeks here the algorithm that most frequently appears at the top of the ranking, which not necessarily means that it is the best quality all the time. In the example of Figure 4, if one would sort by the $\left[L_{dist}\right]$ mean alone, the Li-Tam algorithm would be the top-ranked, as for Image 2 its $\left[L_{dist}\right]$ is higher than most of the other algorithms, raising its mean value. However, it only appears as the top algorithm for that single image. For most images, Moments is better ranked, indicating that for any given image in such a data set, Moments may provide better results.

The simple mean sorting method is applicable to the first way of assessing the algorithms, as the aggregated images have very similar features (capturing device and print type). As for the second way, the different printing types are aggregated to give an overall result for each device, increasing the variability and making the ranking summation more appropriate.

## 3. Choosing the Best Channel

The recent paper [17] showed that there may be a quality difference in feeding a binarization algorithm with the original color image, its grayscale equivalent (using the luminance formula), or the red, green or blue channel. That fact is important, as having one of the input channels as the best-quality result would save processing space and, consequently, processing time, while the grayscale image demands extra processing time, which may be significant for the faster algorithms. Ideally, one would analyze the best channel for each different type of image; however, for the sake of simplicity, in this study, only the input channel which provided the best PL summation ranking was chosen for each algorithm. In several cases, there was a nearly equal quality result between the red or blue channels and the color image. In some other cases, providing a single channel actually increased the final quality and the channel that more often provided better quality was the red channel. Thus, whenever an algorithm yields similar quality results having the full color image and one of the channels as input, the red channel is chosen, as that often means less processing time and space.

Six of the best-ranked algorithms are presented in Table 3 with their respective average PL and the score of the ranking summation, stressing that the lower the score, the better the algorithm. The algorithm by Singh was one of the few that the blue channel offered better results. Among the best algorithms, Sauvola was the one with the greatest difference between applying a single channel or the original color image.

## 4. Results

For each device model, with the in-built strobe-flash on and off, the binarization algorithms were evaluated in two contexts: clustering by the specific image characteristics; and aggregating the whole dataset (global evaluation). In all results, the letter after the original algorithm indicates the version of the image used: R—red; G—green; B—blue; L—luminance; C—original color image. The mean processing time was taken to evaluate the order of magnitude of the time complexity of the algorithms, thus minor time differences are not relevant to this study. The grayscale conversion time was not considered here.

Table 4 presents the results for each device using the ranking summation strategy. YinYang22 and Michalak21a are often among the top 5 for any of the tested devices. For Samsung Note 10+, only HuangUNet presented significant improvement using a single channel other than red. For Samsung S21 Ultra 5G, ElisaTV presented good results compared to recent efficient ones such as YinYang22. For Motorola G9, Michalak21a would be recommended either with flash on or off, due to high quality and low processing time. For Samsung A10S, Michalak21a would also be the one recommended. For Samsung S20, even the most classical algorithm (Ostu) could properly binarize photos taken with flash on. It is important to notice that Dataset 2 has less complex images than Dataset 1. For Apple iPhone SE 2 and flash on, which also used Dataset 2, Otsu again appeared as recommended.

The detailed results for each device are presented in Table 5, Table 6, Table 7 and Table 8. The quality-time criteria was used (Figure 3), as the variation in image characteristics is lower, and thus the standard variation is small enough to allow a fair assessment. It is important to remark that the standard deviation (SD) of the $\left[L_{dist}\right]$ for the Laser and Deskjet dataset was, for all the top 5 and nearly all the other algorithms, approximately 0.04, and for book dataset it was of 0.01, being in some cases close to zero. Only for devices Samsung S21 Ultra 5G and Samsung Note 10+ there was a more significant variation, with the standard deviation varying from 0.1 to 0.3. Those results demonstrate that the top five algorithms for all test datasets provide excellent binarization results for OCR in general.

The PL standard variation was higher due to a higher variation of the $P_{err}$ measure, which is part of it. For all devices, the SD of the Deskjet and Laser dataset was approximately 4.00, while for book dataset, it was under 1 for the devices Motorola G9, Samsung S20, Samsung A10S and between 1 and 3 for devices Samsung Note 10+, Apple iPhone SE 2, Samsung S21 Ultra 5G. The overall quality perceived by visually inspecting the resulting images produced by the top-ranked algorithms is good.

In order to choose the most suitable algorithm for some specific application, the first thing to consider is the intrinsic characteristics of the printing, as different types of ink and printing methods imply entirely different recommendations, as shown in the tables of results. If the document was printed with a deskjet device, it is recommended to check whether the strobe flash should be on or off prior to the image acquisition. After that, the binarization algorithm with the best quality-time balance must be applied. If an application has no significant time constraint, but the quality is so crucial that even a small amount of lost information is not acceptable, one should choose the top quality-time. However, if the image binarization is part of an embedded application, its processing time is a crucial factor, thus the best quality-time trade-off must be chosen.

Two quality measures were used to support the decision of two types of applications: OCR transcription and printing, archiving or transmission through computer networks. For the first application (OCR transcription), the $\left[L_{dist}\right]$ measure should be used, as it does not take into account the visual quality, but only the OCR precision, giving the algorithms with the best chance to provide the best transcription possible. For the second application, the visual quality is also important, thus the PL measure is used, which allows the choice of the best algorithm for OCR transcription and, at the same time, for printing or transmitting.

In general, keeping the strobe flash on or off does not imply any significant difference in the quality of the best-ranked algorithms; however, in most cases, the set of recommended algorithms varies across the devices. For instance, using Samsung S21 Ultra 5G, the algorithms recommended for deskjet printed documents are similar if one keeps the flash on or off, but they are completely different for book offset-printed documents. The same happens for most other devices, either using the $\left[L_{dist}\right]$ or the PL measure when comparing different setups. This fact highlights the importance of considering as many more algorithms as possible, as in some cases, one algorithm that offers excellent results with one configuration may have totally different results with a different set of capturing conditions, devices and setup.

In the results table for $\left[L_{dist}\right]$ measure, the first red line represents the performance of applying the original color image directly on Google Vision OCR without prior binarization. In most cases, the results are equivalent to the performance of providing a binary image. However, for the Motorola G9 and Apple iPhone SE 2, no OCR output is given for most of the captured images. The standard deviation in all cases was nearly zero, which means there were almost no results for the images. This shows that general-purpose OCR engines can be greatly improved when provided with a clean binary image.

**Figure 5:**
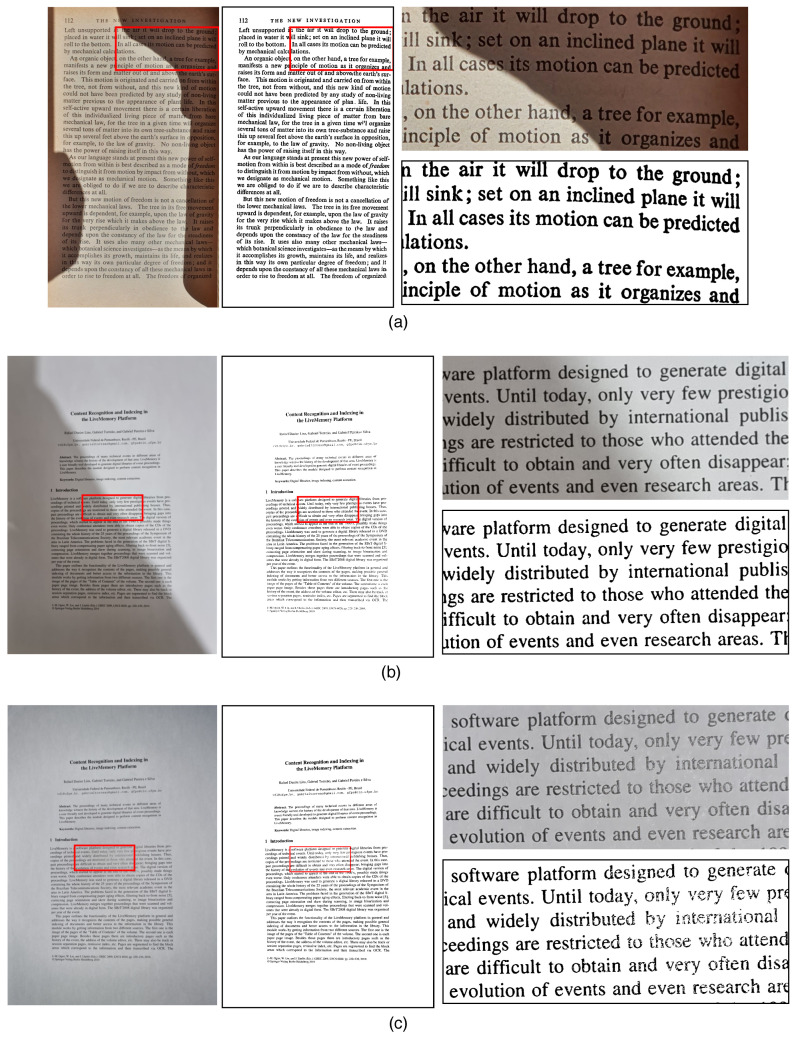
Dataset 1 example images. The red boxes indicates the crop region for the zoomed example next to each image. (**a**) Samsung Note 10+, book offset page, strong natural light, flash off with strong shadow, binarized by HuangUNet-B; (**b**) Samsung S21, laser printed, artificial light, medium shadow, flash off, binarized by Wolf-R; (**c**) Same as (**b**), but with flash on and binarized by YinYang22-R.

In several cases, the recommended algorithms for OCR ($\left[L_{dist}\right]$) match the recommendations using the PL measure with the same input channel or a different one. For instance, using Wolf-R to binarize laser documents with flash off captured by the Samsung S21 Ultra 5G yields not only excellent OCR results, but also good visual quality images. If one checks the example binary image using that algorithm at Figure 5b, it is possible to observe how well this algorithm went, generating a clear binary image with nearly no noise.

It is remarkable how classical global algorithms such as Otsu, dSLR and WAN were quality-time top-ranked, but only when using the in-built strobe flash on. This happened because the flash was sufficient to diminish the shadows and allow those global algorithms to work well and highlights that very simple and fast algorithms can still be used for uniform images, even if photographed in different places and by different smartphones.

**Figure 6:**
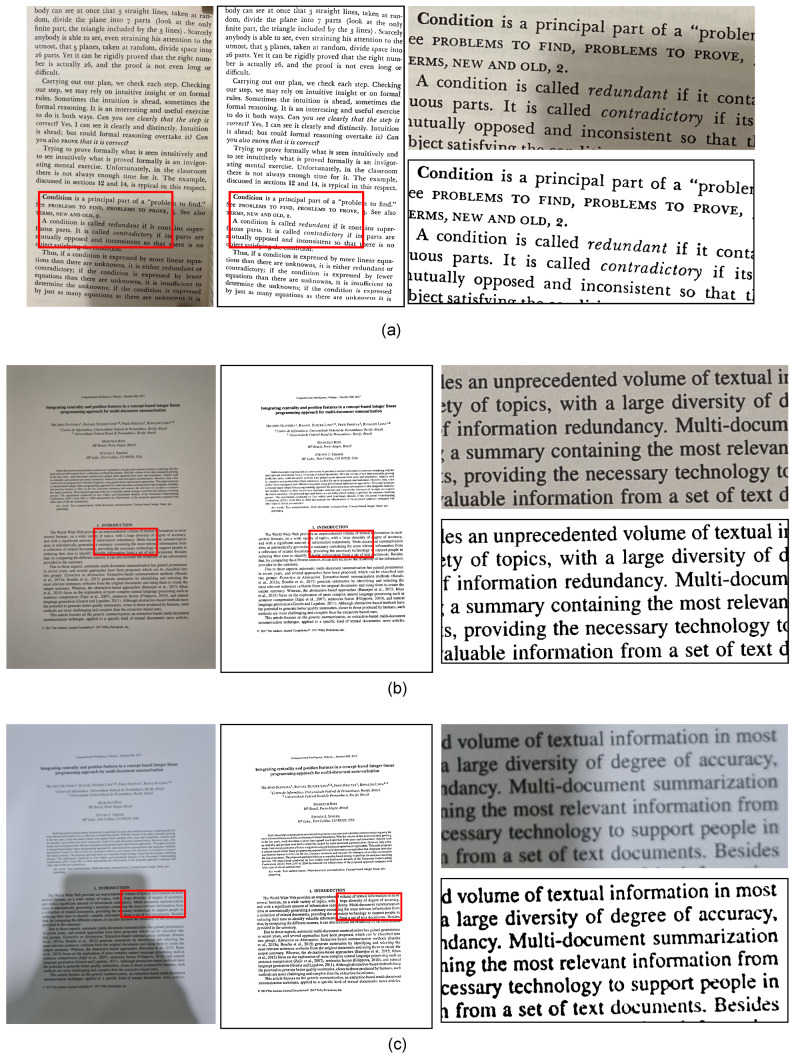
Dataset 2 example images. The red boxes indicates the crop region for the zoomed example next to each image. (**a**) Apple iPhone SE 2, book offset page, artificial light, flash off with medium shadow; (**b**) Samsung S20, deskjet printed, artificial light, medium shadow, flash off; (**c**) Same as (**b**), but with flash on, note that on deskjet printed pages no flash reflex interfere on the photo.

Figure 5 and Figure 6 present some example images. For each input color image, one of the most recommended algorithms is used, according to the global ranking of Table 4. The cropped portion of the image shows the critical regions where shadows and the flash light reflex can be noticed. For nearly all images, an almost perfect binary image was generated. Only in Figure 5c it is possible to see some noise due to the strong flash light reflected on the printed laser page. The laser printing process creates a surface that reflects more light than other types of printing, thus even on the color image, some pixels inside the text stroke are very close to the background ones, making it almost impossible to generate a perfect binary image. No algorithm tested here did better than that, which highlights a possible problem to be solved by future proposals.

## 5. Conclusions

Document binarization is a key step in many document processing pipelines, demanding for quality and time performance. This paper analyses the performance of 68 binarization algorithms in images acquired using six different models of smartphones from three different manufacturers, widely used today. The quality, size and processing time of the binarization algorithms are assessed. A novel quality measure is proposed that combines the Levenshtein distance with the overall visual quality of the binary image. The mean compression rate of the TIFF G4 file with RLE compression was also analyzed; it also provides a quality analysis as the quantity of salt-and-pepper noise in the final image degrades file compression performance.

The results were presented through two perspectives: a detailed evaluation considering the device, the in-built strobe flash state (on or off), and the printing technology (deskjet, laser, or offset); a device-based evaluation considering the visual quality and compressed binary image file size.

Several conclusions may be drawn from the presented results:

1. Keeping the strobe flash on or off may not imply in a better quality image, but one needs to make the right choice of the binarization algorithm in order to have the best monochromatic image.
2. The ranking order is nearly completely different through all the different possible setups, thus it reinforces the claim that no binarization algorithm is good for all document images.
3. The quality of the images yielded by the top-rated algorithms with the offset-printed documents (book) dataset is almost perfect if considering the OCR transcriptions precision.
4. In several cases, as for Apple iPhone SE 2, some global algorithms had the best performance. They are much faster than the newer algorithms and, in some rare cases, even generate cleaner images (better PL).
5. Even when not in the top rank, newer algorithms such as Michalak or YinYang algorithms and their variants are dominant in the results. It is important to stress that they were developed having as target photographed documents, while most of the other algorithms, overall the global ones, were developed aiming at the scanned document images.
6. If the compression rate is a priority, YinYang22, with any of the input versions of the image, would be the most recommended algorithm overall, as it offers the best compression rates while maintaining high quality.
7. If processing time is a priority, Michalak21a with the red channel would be the most recommended algorithm overall, as it requires a small processing time, comparable to one of the classical algorithms, while providing high-quality binary images.
8. This paper also shows that the PL measure provides a better overall quality evaluation of binarization algorithms.
9. Analyzing the TIFF G4 compression rate with RLE has also proved valuable, as, on several occasions, two algorithms provided similar quality results, but one may be two times more efficient in this compression scheme.
10. None of the tested algorithms could perfectly binarize the regions of the laser-printed documents in which the strobe flash (whenever on) created a strong noise in the central region of the image, which suggests that such a set-up should be avoided when photographing laser printed documents.

The recent paper [18] changes the outlook from the document to the device, in such a way that if one had to in-built one binarization algorithm in an embedded application handling document images, which would that be? That algorithm would have to be light and fast enough to yield good quality-space-time performance. Following that approach and looking at Table 4, one could recommend the following algorithms for each device:

**Samsung Note 10+:**  YinYang22-R, Yasin-R, Michalak-R or HuangUNet-B.
**Samsung S21 Ultra 5G:**  ElisaTV-R, YinYang22-R, Michalak21a-R or Singh-B.
**Motorola G9:**  Michalak21a-R, Michalak-R, YinYang-R, ElisaTV-R.
**Samsung A10S:**  Michalak21a-R, YinYang22-R, Wolf-R, Singh-B.
**Samsung S20:**  Michalak21c-R, Michalak-R, YinYang22-R, YinYang-R.
**Apple iPhone SE 2:**  Yasin-R, YinYang22-R, YinYang21-R, Singh-B.

No doubt the list above may suffer variations as visual inspection carries some degree of subjectivity amongst time performances of around the same order of magnitude.

The authors of this paper recently became aware of the reference [73], in which the authors look at the impact on binarization of the color-to-gray conversion algorithms. Besides the binarization performance of the color-to-gray CIE Y (International Commission on Illumination luminance channel) conversion algorithm (assessed here), reference [73] looks at five other algorithms. It proposes two new schemes focusing on the quality of the final monochromatic image and makes a global assessment of scanned documents. The analysis of the performance of such color-to-gray conversion algorithms on photographed documents is left for further work.

Another important point also left as line for further work is setting in-built the strobe flash in auto mode, which means that the device itself will decide, depending overall on the quantity of light in the environment, if the flash will be activated or not.

## Figures and Tables

**Table 1:** Summary of devices camera specifications.

	Samsung N10+	Samsung S21U	Moto. G9 Plus	Samsung A10	Samsung S20	iPhone SE2
Megapixels	16	12	12	13	12	12
Aperture	F 1.5–2.4	F 1.5	F 1.8	F 1.9	F 1.8	F 1.8
Sensor size	1/2.55 inch	1/1.8 inch	1/1.73 inch	-	1/2.55 inch	1/3 inch
Pixel size	-	1.4 μm	1.4 μm	-	1.4 μm	1.4 μm
Release year	2019	2021	2020	2020	2020	2020
Camera Count	3	4	4	2	3	1

**Table 2:** Tested binarization algorithms.

Method	Year	Category	Description
Percentile [20]	1962	Global threshold	Based on partial sums of the histogram levels
Triangle [21]	1977	Global threshold	Based on most and least frequent gray level
Otsu [22]	1979	Global threshold	Maximize between-cluster variance of pixel intensity
IsoData [23]	1980	Global threshold	IsoData clulstering algorithm applied to image histogram
Pun [24]	1981	Global threshold	Defines an anisotropy coefficient related to the asymmetry of the histogram
Johannsen-Bille [25]	1982	Global threshold	Minimizes formula based on the image entropy
Kapur-SW [26]	1985	Global threshold	Maximizes formula based on the image entropy
Moments [27]	1985	Global threshold	Aims to preserve the moment of the input picture
Niblack [28]	1985	Local threshold	Based on window mean and the standard deviation
Bernsen [29]	1986	Local threshold	Uses local image contrast to choose threshold
MinError [30]	1986	Global threshold	Minimum error threshold
Mean [31]	1993	Global threshold	Mean of the grayscale levels
Shanbhag [32]	1994	Global threshold	Improves Kapur-SW by viewing the two pixel classes as fuzzy sets
Huang [33]	1995	Global threshold	Minimizes the measures of fuzzines
Yen [34]	1995	Global threshold	Multilevel threshold based on maximum correlation criterion
RenyEntropy [35]	1997	Global threshold	Uses Renyi’s entropy similarly as Kapur-SW method
Sauvola [36]	1997	Local threshold	Improvement on Niblack
Li-Tam [37]	1998	Global threshold	Minimum cross entropy
Wu-Lu [38]	1998	Global threshold	Minimizes the difference between the entropy of the object and the background
Mello-Lins [13]	2000	Global threshold	Uses Shannon Entropy to determine the global threshold. Possibly the first
			to properly handle back-to-front interference
Wolf [39]	2002	Local threshold	Improvement on Sauvola with global normalization
ISauvola [40]	2004	Local threshold	Uses image contrast in combination with Sauvola’s binarization
Ergina-Global [41]	2005	Global threshold	Average color value and histogram equalization
Ergina-Local [42]	2006	Local threshold	Detects where to apply local thresholding after a applying a global one
Intermodes [43]	2006	Global threshold	Smooth histogram until only two local maxima
Minimum [43]	2006	Global threshold	Variation of Intermodes algorithm
dSLR [44]	2006	Global threshold	Uses Shannon entropy to find a global threshold
Bradley [45]	2007	Local threshold	Adaptive thresholding using the integral image of the input
Nick [46]	2009	Local threshold	Adapts Niblack based on global mean
ElisaTV [47]	2010	Local threshold	Background estimation and subtraction
Lu-Su [48]	2010	Edge based	Local thresholding near edges after background removal
Bataineh [49]	2011	Local threshold	Based on local and global statistics
Singh [50]	2011	Global threshold	Uses integral sum image prior to local mean calculation
Howe [51]	2013	CRF Laplacian	Unary term and pairwise Canny-based term
Su-Lu [52]	2013	Edge based	Canny edges using local contrast
iNICK [53]	2017	Local threshold	Adaptively sets k in Nick method based on the global standard deviation
CNW [54]	2018	Local threshold	Combination of Niblack and Wolf’s algorithm
DocDLinkNet [55]	2018	Deep Learning	D-LinkNet architecture with document image patches
Gattal [56]	2018	Clustering	Automatic Parameter Tuning of K-Means Algorithm
Jia-Shi [57]	2018	Edge based	Detecting symmetry of stroke edges
Robin	2018	Edge based	U-net model trained with several datasets
			(https://github.com/masyagin1998/robin, accessed on 17 January 2023)
WAN [58]	2018	Global threshold	Improves Sauvola’s method by shifting up the threshold
Akbari_1 [59]	2019	Deep Learning	Segnet network architecture fed by multichannel images (wavelet sub bands)
Akbari_2 [59]	2019	Deep Learning	Variation of Akibari_1 with multiple networks
Akbari_3 [59]	2019	Deep Learning	Variation of Akibari_1 where fewer channels are used
CLD [60]	2019	Local threshold	Contrast enhancement followed by adaptive thresholding and artifact removal
Calvo-Zaragoza [61]	2019	Deep learning	Fully convolutional Encoder–decoder FCN with residual blocks
DeepOtsu [62]	2019	Deep Learning	Neural networks learn degradations and global Otsu generates binarization map
DocUNet [9]	2019	Deep Learning	Hybrid pyramid U-Net convolutional network fed with morphological
			bottom-hat transform enhanced document images
Michalak21${}_{a}$ [63]	2019	Image Processing	Downsample image to remove low-frequency information and apply Otsu
Michalak21${}_{b}$ [64]	2019	Image Processing	Equalize illumination and contrast, apply morphological dilatation
			and Bradley’s method
Michalak21${}_{c}$ [65]	2019	Local threshold	Average brightness corrected by two parameters to apply local threshold
Michalak [63]	2019	Image Processing	Downsample image to remove low-frequency information and apply Otsu
Yasin [9]	2019	Image Processing	Gradient descent optimization followed by Otsu thresholding
Yuleny [9]	2019	Shallow ML	A XGBoost classifier is trained with features generated from Otsu, Niblack,
			Sauvola, Su and Howe algorithms
DiegoPavan [66]	2020	Deep Learning	Downscale image to feed a DE-GAN network
DilatedUNet [11]	2020	Deep Learning	Downsample to smooth image and use a dilated convolutional layer to
			correct the feature map spatial resolution
YinYang [11]	2020	Image Processing	Detect background with median of small overllaping windows, extract it and
			apply Otsu
YinYang21 [11]	2020	Image Processing	A faster and more effective version of YinYang algorithm
DE-GAN [66]	2020	Deep Learning	Uses a conditional generative adversarial network
Gosh [67]	2021	Clustering	Clustering applied to a superset of foreground estimated by Niblack’s algorithm
HuangBCD [10]	2021	Deep Learning	BCD-Unet based model to binarize and combine image patches
HuangUNet [10]	2021	Deep Learning	Unet based model binarize and combine image patches
Vahid [10]	2021	Deep Learning	A pixel-wise segmentation model based on Resnet50-Unet
HBUT [68]	2021	Image Processing	Morphological operations using minimum entropy-based stroke width transform and Laplacian energy-based segmentation
DPLinkNet [69]	2021	Deep Learning	Fully dilated convolutional network using atrous convolutions
Vahid22 [16]	2022	Deep Learning	Pixel-wise segmentation combining a CNN with a transformer model
YinYang22 [16]	2022	Image Processing	Uses maximum color occurrence to detect and subtract background, then normalize and apply Otsu

**Table 3:** Example of the choice of a channel with some of the best algorithms.

Team	Best Channel	Best Channel		Color Image		Luminance	
		Score	Mean PL	Score	Mean PL	Score	Mean PL
Michalak21a	Red	632	96.10	817	96.11	727	96.16
YinYang22	Red	649	93.03	825	93.42	687	93.42
Singh	Blue	658	96.14	846	95.42	694	94.98
Wolf	Red	635	94.53	844	93.09	687	95.07
Sauvola	Red	644	93.37	897	90.37	650	93.03

**Table 4:** Overall results by capturing device sorted according to the ranking summation criterion.

FLASH OFF						FLASH ON				
**Rank**	**Algorithm**	**Score**	PL	${\mathrm{CR}}_{G4}$	**Time (s)**	**Algorithm**	**Score**	PL	${\mathrm{CR}}_{G4}$	**Time (s)**
Dataset 1										
	Samsung Note 10+									
1	HuangUNet-B	245	96.46	75.22%	58.67	YinYang22-R	261	96.43	79.99%	5.85
2	YinYang22-R	263	96.25	80.25%	6.50	HuangUNet-B	266	96.37	74.79%	58.05
3	Yasin-R	263	96.18	65.60%	1.90	ElisaTV-R	315	95.79	47.36%	8.82
4	iNICK-R	266	96.11	49.26%	3.46	HuangBCD-R	321	96.04	74.88%	249.90
5	Michalak-R	283	96.22	49.17%	0.06	Yasin-R	329	95.65	64.91%	1.76
	Samsung S21 Ultra 5G									
1	ElisaTV-R	235	96.30	47.81%	10.38	YinYang22-R	273	91.36	80.20%	5.54
2	YinYang22-R	243	96.13	80.05%	6.36	Michalak21a-R	276	95.98	48.40%	0.04
3	Yasin-R	265	95.95	65.02%	1.78	Singh-B	285	95.45	76.03%	0.34
4	Michalak21a-R	269	91.51	48.02%	0.05	Nick-R	286	95.26	76.07%	0.16
5	Singh-B	289	94.34	75.68%	0.32	ElisaTV-R	310	95.74	48.06%	10.07
Dataset 2										
	Motorola G9									
1	Michalak21a-R	218	96.92	47.51%	0.05	Gattal-R	138	97.23	63.09%	53.09
2	ElisaTV-R	230	96.75	45.83%	12.47	Michalak21a-R	150	97.26	47.83%	0.05
3	Michalak-R	230	96.88	47.51%	0.05	YinYang-R	164	97.23	78.48%	1.81
4	YinYang21-R	231	96.83	69.14%	1.71	ElisaTV-R	181	97.18	47.18%	12.21
5	Michalak21c-R	231	96.90	46.71%	1.48	YinYang21-R	214	97.12	69.33%	1.64
	Samsung A10S									
1	YinYang22-R	232	97.08	80.84%	4.63	Wolf-R	140	97.24	75.19%	0.16
2	Michalak21a-R	247	97.03	44.06%	0.03	Singh-B	147	97.23	75.19%	0.24
3	Michalak-R	248	97.01	44.13%	0.03	Yasin-R	149	97.26	62.78%	1.30
4	Michalak21c-R	265	96.99	44.07%	0.84	Michalak21a-R	155	97.17	44.03%	0.03
5	YinYang21-R	282	96.85	66.65%	1.08	Nick-R	174	97.21	75.11%	0.11
	SamsungS20									
1	Michalak21c-R	199	97.00	47.97%	1.09	Gattal-R	170	97.20	63.78%	52.14
2	Michalak-R	216	96.86	48.16%	0.04	Otsu-R	189	97.11	75.93%	0.02
3	Michalak21a-R	230	96.88	48.13%	0.04	YinYang-R	210	97.08	77.29%	1.42
4	Bradley-R	251	96.82	76.34%	0.29	YinYang22-R	226	97.13	81.39%	5.07
5	YinYang-R	266	96.82	78.03%	1.45	Li-Tam-R	246	97.04	75.89%	0.12
	Apple iPhone SE 2									
1	Yasin-R	156	95.44	63.18%	1.59	Otsu-R	192	97.03	75.11%	0.01
2	Sauvola-R	162	96.93	75.49%	0.14	YinYang22-R	211	96.94	81.19%	5.29
3	Singh-B	163	96.94	75.47%	0.23	Yasin-R	229	96.89	62.80%	1.40
4	YinYang22-R	167	96.87	81.32%	5.51	YinYang21-R	235	96.88	67.15%	1.14
5	Nick-R	173	96.90	75.46%	0.14	Gattal-R	235	96.88	62.28%	51.36

**Table 5:** Summary of results with PL measure and flash OFF sorted according to the quality-time criteria.

	DESKJET			LASER			BOOK		
Rank	Algorithm	PL	Time (s)	Algorithm	PL	Time (s)	Algorithm	PL	Time (s)
	Dataset 1—Flash OFF								
	Samsung Note 10+								
1	iNICK-R	96.47	3.48	Sauvola-R	96.59	0.19	Vahid22-C	98.41	29.22
2	Sauvola-R	96.07	0.19	Nick-R	96.58	0.19	HuangUNet-B	98.18	50.22
3	Yasin-R	95.99	1.77	iNICK-R	96.57	3.49	CNW-R	97.97	3.60
4	Nick-R	95.88	0.19	Yasin-R	96.50	1.94	DPLinkNet-C	97.87	9.10
5	Singh-B	95.78	0.40	ElisaTV-R	96.50	11.66	DocDLink-C	97.81	7.01
	Samsung S21 Ultra 5G								
1	Sauvola-R	96.59	0.19	Wolf-R	96.75	0.26	Michalak-R	97.78	0.04
2	iNICK-R	95.89	3.43	Nick-R	96.54	0.19	CNW-R	97.75	3.37
3	Wolf-R	95.81	0.25	Singh-B	96.45	0.38	ElisaTV-R	97.65	8.73
4	Singh-B	95.66	0.37	Yasin-R	96.22	1.85	Vahid22-C	97.45	29.14
5	Nick-R	95.62	0.18	iNICK-R	96.14	3.49	Jia-Shi-R	97.44	18.45
	Dataset 2—Flash OFF								
	Motorola G9								
1	Nick-R	96.20	0.21	YinYang21-R	96.52	1.67	Michalak21b-R	99.10	3.13
2	iNICK-R	95.63	3.53	YinYang-R	96.51	1.74	Michalak21c-R	99.06	1.48
3	YinYang21-R	95.56	1.73	iNICK-R	96.46	3.50	CNW-R	99.01	3.55
4	Singh-B	95.48	0.51	Nick-R	96.34	0.20	Michalak-R	98.99	0.05
5	Yasin-R	95.44	2.13	Michalak21a-R	96.28	0.05	DPLinkNet-C	98.86	11.86
	Samsung A10S								
1	Sauvola-R	96.31	0.12	YinYang22-R	96.70	4.59	ISauvola-R	99.14	0.31
2	Singh-B	96.23	0.26	ElisaTV-R	96.55	7.39	Michalak21c-R	98.97	0.84
3	Nick-R	96.15	0.12	YinYang-R	96.51	1.08	Michalak-R	98.80	0.03
4	Yasin-R	95.90	1.30	Michalak21a-R	96.41	0.03	Vahid22-C	98.80	17.47
5	iNICK-R	95.80	3.27	YinYang21-R	96.36	1.04	WAN-R	98.77	0.78
	Samsung S20								
1	Nick-R	96.10	0.15	YinYang-R	96.10	1.41	Michalak21c-R	99.10	1.04
2	Singh-B	95.83	0.34	Michalak21c-R	96.07	1.14	DocUNet-L	99.07	45.50
3	iNICK-R	95.63	3.35	Michalak21a-R	95.98	0.04	Michalak-R	99.06	0.04
4	Yasin-R	95.31	1.63	Bradley-R	95.98	0.31	ISauvola-R	99.05	0.38
5	YinYang-R	95.19	1.37	Michalak-R	95.95	0.04	Bradley-R	99.04	0.28
	Apple iPhone SE 2								
1	Yasin-R	95.51	1.67	Yasin-R	96.65	1.60	Singh-B	98.70	0.17
2	Nick-R	95.40	0.14	YinYang22-R	96.52	6.02	YinYang21-R	98.66	1.11
3	Sauvola-R	95.35	0.15	ElisaTV-R	96.50	7.38	Sauvola-R	98.59	0.12
4	YinYang22-R	95.31	5.76	Nick-R	96.37	0.16	Wolf-R	98.53	0.17
5	iNICK-R	95.30	3.31	Sauvola-R	96.28	0.16	Nick-R	98.42	0.12

**Table 6:** Summary of results with PL measure and flash ON state sorted according to the quality-time criteria.

	DESKJET			LASER			BOOK		
Rank	Algorithm	PL	Time (s)	Algorithm	PL	Time (s)	Algorithm	PL	Time (s)
	Dataset 1—Flash ON								
	Samsung Note 10+								
1	Sauvola-R	96.25	0.19	YinYang22-R	96.69	6.35	HuangUNet-B	97.62	48.25
2	Yasin-R	96.07	1.98	ElisaTV-R	96.68	11.88	Calvo-Z-R	97.59	1.26
3	Nick-R	96.01	0.19	Yasin-R	96.65	1.82	DocDLink-C	97.29	6.55
4	Singh-B	95.94	0.37	Sauvola-R	96.60	0.20	DocUNet-L	97.27	39.87
5	Yen-CC-C	95.92	0.16	YinYang21-R	96.52	1.55	Vahid22-C	97.24	27.96
	Samsung S21 Ultra 5G								
1	Nick-R	96.11	0.18	Singh-B	96.66	0.41	HuangBCD-R	98.12	202.48
2	Singh-B	96.09	0.40	Nick-R	96.58	0.18	WAN-R	97.78	0.87
3	Wolf-R	95.68	0.25	Michalak21a-R	96.02	0.05	HuangUNet-B	97.65	47.00
4	Michalak21a-R	95.27	0.05	Yasin-R	95.97	1.91	CNW-R	97.62	3.35
5	Yasin-R	95.27	1.80	YinYang21-R	95.91	1.55	DocDLink-C	97.48	6.28
	Dataset 2—Flash OFF								
	Motorola G9								
1	Sauvola-R	96.66	0.22	Nick-R	96.74	0.20	Michalak21a-R	99.29	0.05
2	Nick-R	96.08	0.21	YinYang-R	96.62	1.69	ElisaTV-R	99.28	11.42
3	Singh-B	95.81	0.49	Gattal-R	96.60	53.34	Bradley-R	99.24	0.35
4	Wolf-R	95.57	0.29	Singh-B	96.58	0.45	Michalak21c-R	99.15	1.30
5	YinYang-R	95.56	1.83	YinYang21-R	96.44	1.59	Michalak-R	99.06	0.05
	Samsung A10S								
1	Sauvola-R	96.23	0.12	Nick-R	96.40	0.11	Wolf-R	99.46	0.16
2	Yasin-R	95.68	1.25	Yasin-R	96.38	1.27	Michalak21c-R	99.41	0.80
3	ElisaTV-R	95.62	5.95	YinYang-R	96.18	1.05	Michalak21a-R	99.35	0.03
4	Nick-R	95.56	0.12	Wolf-R	96.12	0.16	Singh-B	99.32	0.23
5	Singh-B	95.56	0.25	Singh-B	96.12	0.25	YinYang22-R	99.20	4.47
	Samsung S20								
1	Shanbhag-R	96.36	0.13	Sauvola-R	96.67	0.16	Ergina${}_{L}$-L	99.42	0.56
2	Nick-R	95.77	0.15	Yasin-R	96.66	1.59	Michalak21c-R	99.36	0.95
3	Singh-B	95.57	0.33	Otsu-R	96.57	0.02	Michalak21a-R	99.35	0.04
4	Gattal-R	95.30	52.04	YinYang22-R	96.51	5.27	Bradley-R	99.35	0.26
5	Sauvola-R	95.26	0.16	Gattal-R	96.49	52.64	Ergina${}_{G}$-L	99.28	0.42
	Apple iPhone SE 2								
1	ElisaTV-R	96.11	3.18	Otsu-R	96.57	0.02	YinYang21-R	98.74	1.09
2	Gattal-R	95.93	51.76	Nick-R	96.55	0.15	Ergina${}_{G}$-L	98.60	0.36
3	Li-Tam-R	95.87	0.12	ElisaTV-R	96.54	4.07	YinYang-R	98.58	1.34
4	Nick-R	95.83	0.15	Singh-B	96.53	0.26	Ergina${}_{L}$-L	98.56	0.49
5	Singh-B	95.79	0.26	YinYang22-R	96.51	5.51	YinYang22-R	98.56	4.26

**Table 7:** Summary of results with Ldist measure and flash OFF sorted according to the quality-time criteria. Note that Google Vision (in red) is not a binarization algorithm, but an OCR platform.

	DESKJET			LASER			BOOK		
**Rank**	**Algorithm**	$\left[L_{\mathrm{dist}}\right]$	**Time (s)**	**Algorithm**	$\left[L_{\mathrm{dist}}\right]$	**Time (s)**	**Algorithm**	$\left[L_{\mathrm{dist}}\right]$	**Time (s)**
	Dataset 1—Flash OFF								
	Samsung Note 10+								
0	Google Vision	0.971	–	Google Vision	0.971	–	Google Vision	0.984	–
1	HuangUNet-B	0.971	64.271	HuangUNet-B	0.971	64.329	iNICK-R	0.990	3.421
2	Michalak-R	0.970	0.051	Michalak-R	0.970	0.051	Vahid22-C	0.990	29.224
3	Nick-R	0.970	0.188	Michalak21a-R	0.970	0.052	Singh-B	0.988	0.255
4	Sauvola-R	0.970	0.194	Nick-R	0.970	0.188	Yasin-R	0.986	1.967
5	Bradley-R	0.970	0.352	Singh-B	0.970	0.408	HuangUNet-B	0.986	50.216
	Samsung S21 Ultra 5G								
0	Google Vision	0.971	–	Google Vision	0.971	–	Google Vision	0.982	–
1	Jia-Shi-R	0.971	22.391	Wolf-R	0.971	0.259	Niblack-C	0.988	0.133
2	Wolf-R	0.970	0.254	CNW-R	0.971	3.506	ElisaTV-R	0.986	8.726
3	ISauvola-R	0.970	0.453	Jia-Shi-R	0.971	22.470	Michalak-R	0.985	0.038
4	WAN-R	0.970	1.209	Nick-R	0.970	0.187	Bradley-R	0.984	0.266
5	Michalak21c-R	0.970	1.328	Robin-L	0.970	0.979	WAN-R	0.984	0.913
	Dataset 2—Flash OFF								
	Motorola G9								
0	Google Vision	0.000	–	Google Vision	0.000	–	Google Vision	0.001	–
1	Bradley-R	0.968	0.401	iNICK-R	0.970	3.503	WAN-R	0.997	1.226
2	CNW-R	0.968	3.595	ISauvola-R	0.969	0.491	CNW-R	0.997	3.547
3	YinYang22-R	0.968	6.636	YinYang21-R	0.969	1.672	Jia-Shi-R	0.997	23.597
4	Michalak21a-R	0.967	0.055	CNW-R	0.969	3.578	Michalak21a-R	0.996	0.050
5	Michalak-R	0.967	0.056	YinYang22-R	0.969	6.486	Singh-B	0.996	0.391
	Samsung A10S								
0	Google Vision	0.970	–	Google Vision	0.971	–	Google Vision	0.995	–
1	dSLR-R	0.971	0.030	YinYang22-R	0.969	4.588	Michalak21a-R	0.996	0.033
2	WAN-R	0.970	0.795	CNW-R	0.968	3.240	ISauvola-R	0.996	0.308
3	ISauvola-R	0.969	0.294	Vahid22-C	0.968	16.820	WAN-R	0.996	0.776
4	Michalak21c-R	0.969	0.849	Vahid-B	0.968	17.314	Michalak21c-R	0.996	0.838
5	YinYang21-R	0.969	1.050	Michalak21a-R	0.967	0.032	ElisaTV-R	0.996	5.948
	Samsung S20								
0	Google Vision	0.971	–	Google Vision	0.971	–	Google Vision	0.995	–
1	ISauvola-R	0.970	0.376	Michalak21c-R	0.968	1.141	Nick-R	0.996	0.147
2	YinYang22-R	0.970	5.789	CNW-R	0.968	3.441	WAN-R	0.996	0.973
3	Vahid22-C	0.970	21.839	Vahid22-C	0.968	22.565	DE-GAN-G	0.996	3.334
4	WAN-R	0.969	1.032	Michalak-R	0.967	0.043	CNW-R	0.996	3.410
5	Michalak21c-R	0.969	1.103	Bradley-R	0.967	0.307	ElisaTV-R	0.996	8.087
	Apple iPhone SE 2								
0	Google Vision	0.804	–	Google Vision	0.000	–	Google Vision	0.990	–
1	Ergina${}_{G}$-L	0.972	0.409	Otsu-R	0.971	0.017	WAN-R	0.991	0.798
2	Gattal-R	0.972	50.697	WAN-R	0.971	1.027	CNW-R	0.991	3.416
3	Otsu-R	0.971	0.015	DPLinkNet-C	0.971	9.845	Singh-B	0.990	0.173
4	Li-Tam-R	0.971	0.105	Vahid-B	0.971	22.857	Bradley-R	0.990	0.214
5	Moments-R	0.970	0.106	Gattal-R	0.971	50.781	ISauvola-R	0.990	0.312

**Table 8:** Summary of results with Ldist measure and flash ON sorted according to the quality-time criteria. Note that Google Vision (in red) is not a binarization algorithm, but an OCR platform.

	DESKJET			LASER			BOOK		
**Rank**	**Algorithm**	$\left[L_{\mathrm{dist}}\right]$	**Time (s)**	**Algorithm**	$\left[L_{\mathrm{dist}}\right]$	**Time (s)**	**Algorithm**	$\left[L_{\mathrm{dist}}\right]$	**Time (s)**
	Dataset 1—Flash ON								
	Samsung Note 10+								
0	Google Vision	0.971	–	Google Vision	0.971	–	Google Vision	0.984	–
1	DocDLink-C	0.971	8.926	Michalak21b-R	0.970	3.230	Nick-R	0.984	0.134
2	DPLinkNet-C	0.971	12.102	Yasin-R	0.969	1.822	YinYang22-R	0.983	5.227
3	Jia-Shi-R	0.971	23.264	Vahid-B	0.969	29.386	Calvo-Z-R	0.981	1.256
4	DilatedUNet-G	0.971	36.097	HuangUNet-B	0.969	65.967	HuangUNet-B	0.981	48.253
5	Michalak-R	0.970	0.049	Akbari${}_{3}$-L	0.969	79.356	WAN-R	0.979	0.890
	Samsung S21 Ultra 5G								
0	Google Vision	0.971	–	Google Vision	0.971	–	Google Vision	0.983	–
1	ISauvola-R	0.971	0.434	Vahid-B	0.969	27.036	HuangBCD-R	0.987	202.484
2	Michalak21a-R	0.970	0.049	Singh-B	0.968	0.414	Michalak21a-R	0.982	0.037
3	WAN-R	0.970	1.183	Nick-R	0.967	0.181	Singh-B	0.982	0.245
4	CNW-R	0.970	3.502	Michalak21c-R	0.967	1.318	WAN-R	0.982	0.865
5	DocDLink-C	0.970	8.442	Vahid22-C	0.967	38.140	HuangUNet-B	0.982	47.002
	Dataset 2—Flash ON								
	Motorola G9								
0	Google Vision	0.000	–	Google Vision	0.000	–	Google Vision	0.001	–
1	Michalak21a-R	0.971	0.055	Michalak21a-R	0.970	0.053	Vahid-B	0.997	26.296
2	Bataineh-R	0.971	0.153	Michalak-R	0.970	0.053	Yen-CC-C	0.996	0.170
3	Nick-R	0.971	0.209	Bataineh-R	0.970	0.147	Singh-B	0.996	0.360
4	Sauvola-R	0.971	0.216	ISauvola-R	0.970	0.478	Ergina${}_{G}$-L	0.996	0.562
5	Bradley-R	0.971	0.396	WAN-R	0.970	1.314	WAN-R	0.996	1.201
	Samsung A10S								
0	Google Vision	0.967	–	Google Vision	0.971	–	Google Vision	0.997	–
1	ElisaTV-R	0.970	5.952	Michalak21a-R	0.968	0.032	Michalak21a-R	0.998	0.034
2	HuangBCD-R	0.970	171.542	Michalak-R	0.968	0.032	Nick-R	0.998	0.115
3	dSLR-R	0.969	0.025	Bradley-R	0.968	0.218	WAN-R	0.998	0.754
4	Moments-R	0.969	0.026	Singh-B	0.968	0.254	Jia-Shi-R	0.998	15.750
5	Michalak21a-R	0.969	0.032	YinYang22-R	0.968	4.308	HuangUNet-B	0.998	39.811
	Samsung S20								
0	Google Vision	0.967	–	Google Vision	0.971	–	Google Vision	0.997	–
1	Nick-R	0.970	0.154	ISauvola-R	0.970	0.362	Otsu-R	0.997	0.014
2	ISauvola-R	0.970	0.372	YinYang22-R	0.970	5.271	dSLR-R	0.997	0.098
3	CNW-R	0.970	3.419	Bataineh-R	0.969	0.111	Li-Tam-R	0.997	0.098
4	YinYang22-R	0.970	5.221	Jia-Shi-R	0.969	20.096	Wolf-R	0.997	0.186
5	Triangle-C	0.969	0.148	Vahid22-C	0.969	21.402	Bradley-R	0.997	0.257
	Apple iPhone SE 2								
0	Google Vision	0.638	–	Google Vision	0.000	–	Google Vision	0.987	–
1	WAN-R	0.971	0.992	ISauvola-R	0.969	0.347	YinYang21-R	0.991	1.087
2	Otsu-R	0.970	0.016	WAN-R	0.969	0.958	Michalak21b-R	0.991	2.254
3	Michalak-R	0.970	0.041	DE-GAN-G	0.969	3.181	DE-GAN-G	0.991	2.860
4	Bataineh-R	0.970	0.114	YinYang22-R	0.969	5.508	Vahid22-C	0.991	16.958
5	Moments-R	0.970	0.122	DocDLink-C	0.969	7.026	Li-Tam-R	0.990	0.034

## Data Availability

The results presented here made use of the IAPR (International Association on Pattern Recognition) DIB—Document Image Binarization dataset, available at: https://dib.cin.ufpe.br, accessed on 17 January 2023.
